# An Autonomous Vehicle Navigation System Based on Inertial and Visual Sensors

**DOI:** 10.3390/s18092952

**Published:** 2018-09-05

**Authors:** Xingxing Guang, Yanbin Gao, Henry Leung, Pan Liu, Guangchun Li

**Affiliations:** 1 College of Automation, Harbin Engineering University, Harbin 150001, China; gaoyanbin@hrbeu.edu.cn (Y.G.); lgc_67@hrbeu.edu.cn (G.L.); 2 Department of Electrical and Computer Engineering, University of Calgary, Calgary, AB T2N1N4, Canada; leungh@ucalgary.ca; 3 Beijing Institute of Control and Electronic Technology, Beijing 100032, China; liupan003@hrbeu.edu.cn

**Keywords:** autonomous vehicle, strapdown inertial navigation system, inertial-visual fusion method, Kalman Filter

## Abstract

The strapdown inertial navigation system (SINS) is widely used in autonomous vehicles. However, the random drift error of gyroscope leads to serious accumulated navigation errors during long continuous operation of SINS alone. In this paper, we propose to combine the Inertial Measurement Unit (IMU) data with the line feature parameters from a camera to improve the navigation accuracy. The proposed method can also maintain the autonomy of the navigation system. Experimental results show that the proposed inertial-visual navigation system can mitigate the SINS drift and improve the accuracy, stability, and reliability of the navigation system.

## 1. Introduction

The successful use of automated vehicles will have a great impact on human live [1]. It will bring more convenient and easier driving experiences for human. How to offer accurate, stable, and reliable navigation information is pivotal in order to keep an automated vehicle safe. The inertial navigation system (INS) has been widely used for automated vehicle positioning and navigating because of its high autonomy, concealment, continuum, insusceptible climate, and the successive supply of position, velocity, and attitude (PVA) information [2]. The strapdown inertial navigation system (SINS) is simple, small in size, and is convenient in maintenance compared with plat INS (PINS) and is also very popular in vehicle navigation systems [3,4]. However, the random drift error of gyroscope in SINS may lead to serious accumulated navigation errors during the long operation of SINS alone. As a result, how to keep the accuracy of SINS has attracted the attention of many researchers.

There are two ways to improve the accuracy of SINS. One uses an enhanced production technology of IMU such as improving the structure of the gyroscope [5,6,7] and using more advanced materials [8,9] to improve the accuracy of SINS. However, these methods will increase the production cost and make a longer research cycle of IMU. The other way is to combine IMU with other sensors to mitigate the SINS drift. Integrated IMU with Global Navigation Satellite System (GNSS) is a widely used approach to improve the accuracy of SINS [10,11,12]. Map-based navigation is another approach to get high accuracy navigation [13]. Fusing map matching with SINS can also improve the accuracy of vehicle navigation [14]. However, the signals of GNSS and satellite imageries can be jammed easily and are sensitive to weather and environmental conditions [15]. In addition, using GNSS and map matching will cause the navigation system to lose the high autonomy of SINS.

Machine vision is increasingly being used in automated driving. An automated car is usually driven on structural roads [16]. The characteristics of structural roads include clear road markings, a sample background, and obvious geometric shapes. Satzoda et al. propose a drive analysis method using a camera, IMU, and the Global Position System (GPS) to get the position of the vehicle [17]. Vivacqua et al. propose the low-cost sensors approach for accurate vehicle localization and autonomous driving that uses a camera and SINS [18]. However, in these applications, the visual sensor is only used for the lanes detection and the visual information is not fused for navigation. An improved Features from Accelerated Segment Test (FAST) feature extraction based on the Random Sample Consensus (RANSAC) method is proposed to remove the mismatched points in Reference [19]. It uses point feature extraction to improve the accuracy of the navigation system during driving. These studies are all about moving vehicles. There is no study on the inertial and visual integrated navigation system when the vehicle stops. However, stops, such as waiting for the traffic lights, comity pedestrians that are inevitable in automated driving. The navigation information in static state is an important part of navigation systems as well. Thus, it is necessary to study the application of the navigation system at a static situation. Moreover, all these published papers on integrated navigation systems are combined with GNSS and lose the autonomy of navigation systems.

In this paper, a novel integrated navigation method only based on inertial visual sensors is proposed. The camera is not used for lane detection. It is the first time that a combined line feature in the image with SINS, in order to constitute an integrated navigation system, has been presented. Additionally, the feasibility of the proposed method is proved by the static experiment. It lays a theoretical foundation for the research of the proposed method in a dynamic situation. Experimental results show that the proposed inertial-visual integrated navigation system can improve the accuracy and reliability of the navigation system.

## 2. Coordinate Systems and Kalman Filter

### 2.1. The Reference Coordinate Systems

The different coordinate systems in this paper are defined as follows:

- Coordinate: Earth-Centered Initially Fixed (ECIF) orthogonal reference coordinate system;
- *t*-coordinate: Orthogonal reference frame aligned with East-North-Up (ENU) geographic coordinate system;
- *b*-coordinate: Body coordinate system;
- *n*-coordinate: Navigation coordinate system;
- *c*-coordinate: Camera coordinate system;
- *im*-coordinate: Image coordinate system.

### 2.2. Kalman Filter

Kalman Filter (KF) is the most widely used estimation method in inertial navigation systems. For a discrete-time system [20], at $t_{k+1}$, the system equations can be expressed by

(1) $$X_{k+1}=\Phi_{k+1,k}X_{k}+\Gamma_{k}W_{k}\;$$

where $X_{k+1}$ is estimated state vector, $\Phi_{k+1,k}$ is the one-step transfer matrix from $t_{k}$ time point to $t_{k+1}$, $\Gamma_{k}$ is the driven-noise matrix, and $W_{k}$ is system excitation noise vector.

The measurement equation is given by

(2) $$Z_{k+1}=H_{k+1}X_{k+1}+V_{k+1}\;$$

where $Z_{k+1}$ is the measurement vector, $H_{k+1}$ is the measurement matrix, $V_{k+1}$ is the measurement noise vector.

The Kalman Filter includes a one-step state prediction equation, a state estimation equation, a filtering gain equation, a one-step prediction mean square error equation, and an estimated mean square error equation. They are listed as below:

(3) $${\hat{X}}_{k+1,k}=\Phi_{k+1,k}{\hat{X}}_{k}\;$$

(4) $${\hat{X}}_{k+1}={\hat{X}}_{k+1,k}+K_{k}\left(Z_{k}-H_{k}{\hat{X}}_{k+1,k}\right)\;$$

(5) $$K_{k+1}=P_{k+1,k}H_{k}^{T}{\left(H_{k}P_{k+1,k}H_{k}^{T}+R_{k}\right)}^{-1}\;$$

(6) $$P_{k+1,k}=\Phi_{k+1,k}P_{k}\Phi_{k+1,k}^{T}+\Gamma_{k}Q_{k}\Gamma_{k}^{T}\;$$

(7) $$P_{k+1}=\left(I-K_{k}H_{k}\right)P_{k+1,k}{\left(I-K_{k}H_{k}\right)}^{T}+K_{k}R_{k}K_{k}^{T}\;$$

where ${\hat{X}}_{k+1,k}$ denotes the prediction of the state vector from $t_{k}$ to $t_{k+1}$, ${\hat{X}}_{k}$ denotes the prediction of the state vector at $t_{k}$, $K_{k}$ denotes filtering gain matrix, $P_{k+1,k}$ denotes one-step estimated mean square error matrix from $t_{k}$ to $t_{k+1}$, $P_{k+1}$ denotes estimated mean square error matrix, $Q_{k}$ is system noise covariance matrix, and $R_{k}$ denotes measurement noise covariance matrix.

To analyze the problem of the SINS static error model arising in the Kalman Filter, the local-level ENU frame is selected as the navigation frame [21]. The state vector of the system error model is defined as

(8) $$X={\left[\delta L,\delta \lambda ,\delta h,\delta V_{E},\delta V_{N},\delta V_{U},\delta \phi_{x},\delta \phi_{y},\delta \phi_{z},\nabla_{E},\nabla_{N},\nabla_{U},\varepsilon_{E},\varepsilon_{N},\varepsilon_{U}\right]}^{T}\;$$

where L, λ and h denote the local latitude, longitude, and height respectively; δV defines the body velocity vector, δϕ defines the body attitude error, and ∇, ε, denote the accelerometer zero-biases, and the constant gyroscope drifts, respectively; the subscript ${}_{E}$, ${}_{N}$, ${}_{U}$ denotes the projection on the East, North, and Up axis of the *t*-coordinate, respectively.

Equation (1) can be written as

(9) $$X_{k+1}=FX_{k}+W_{k}\;$$

where F defines the system matrix as below

(10) $$F=\left[\begin{array}{ccccc} 0 & F_{1,2} & 0 & 0 & 0\\ 0 & F_{2,2} & F_{2,3} & F_{2,4} & 0\\ F_{3,1} & F_{3,2} & F_{3,3} & 0 & F_{3,5}\\ 0 & 0 & 0 & 0 & 0\\ 0 & 0 & 0 & 0 & 0 \end{array}\right]\;$$

where 0 denotes a 3×3 zeros matrix, the non-zero element in F shown as below:

$$F_{1,2}=\left[\begin{array}{ccc} 0 & 1/R & 0\\ \sec L/R & 0 & 0\\ 0 & 0 & 1 \end{array}\right],\;F_{2,2}=\left[\begin{array}{ccc} 0 & 2\omega_{ie}\sin L & -2\omega_{ie}\cos L\\ -2\omega_{ie}\sin L & 0 & 0\\ 2\omega_{ie}\cos L & 0 & 0 \end{array}\right],\;F_{2,3}=\left[\begin{array}{ccc} 0 & -g & 0\\ g & 0 & 0\\ 0 & 0 & 0 \end{array}\right],\;F_{3,1}=\left[\begin{array}{ccc} 0 & 0 & 0\\ -\omega_{ie}\sin L & 0 & 0\\ \omega_{ie}\cos L & 0 & 0 \end{array}\right],\;F_{3,2}=\left[\begin{array}{ccc} 0 & -1/R & 0\\ 1/R & 0 & 0\\ \tan L/R & 0 & 0 \end{array}\right],\;F_{3,3}=\left[\begin{array}{ccc} 0 & \omega_{ie}\sin L & -\omega_{ie}\cos L\\ -\omega_{ie}\sin L & 0 & 0\\ \omega_{ie}\cos L & 0 & 0 \end{array}\right],$$

and $F_{2,4}=F_{3,5}=C_{b}^{n}$, $C_{b}^{n}$ denotes the translated matrix from the *b*-coordinate to the *n*-coordinate, R defines the Earth radius, g defines the gravitational acceleration in the *n*-coordinate, $\omega_{ie}$ defines the angle velocity of the earth in the *i*-coordinate.

Theoretically, when a vehicle is in the stationary base, the velocity is zero and the attitude is invariable. Thus, velocity error and attitude error in the *n*-coordinate can be selected as measurement elements. Therefore, the measurement vector can be written as below

(11) $$Z_{k}=\left[\begin{array}{c} \delta V_{k}\\ \delta \phi_{k} \end{array}\right]\;$$

Therefore, the system measurement equation from Equation (2) can then be written as

(12) $$Z_{k}=HX_{k}+V_{k}\;$$

where $V_{k}$ is a white noise, and the measurement matrix is written as

(13) $$H=\left[\begin{array}{ccc} 0_{6\times 3} & I_{6\times 6} & 0_{6\times 6} \end{array}\right]\;$$

## 3. Visual Image Processing

In this section, we discuss how to obtain the line feature from the image, and how to translate the line feature to the navigation information, which can be combined with SINS. Firstly, the line feature includes the angle feature parameter θ, which is extracted by the Hough transform [22]. Next, by analyzing the mean of Δθ, we get the relative attitude error of the vehicle when it stops. Then, it projected the relative attitude error from the *im*-coordinate to the *b*-coordinate. Thus, the relative attitude error can be combined with the attitude error of SINS via KF.

**Figure 1:**
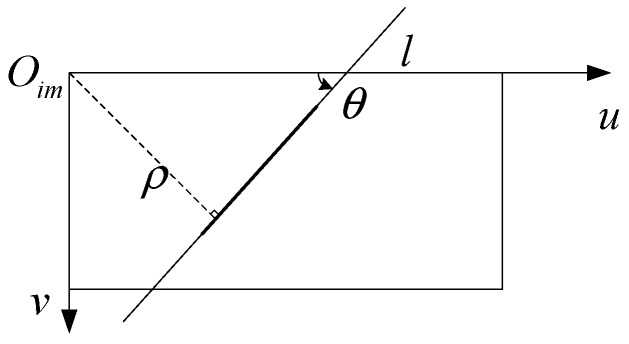
The line representation in the image.

In Figure 1, the line feature extracted by the Hough transform in image processing is given by [22]

(14) vcosθ+usinθ−ρ=0 

where $O_{im}$, u, and v denote the origin point and axis of the image plane, and the axis of the image plane, θ denotes the angle of the line and axis u,θ∈[−π/2,π/2), ρ denote the distance of line and $O_{im}$.

It is important to transform the line feature in the *n*-coordinate as a navigation parameter for fusing image information into the navigation system. As per the discussion below, we can obtain a related attitude error, which can be combined with SINS, from the angle feature parameter θ.

**Figure 2:**
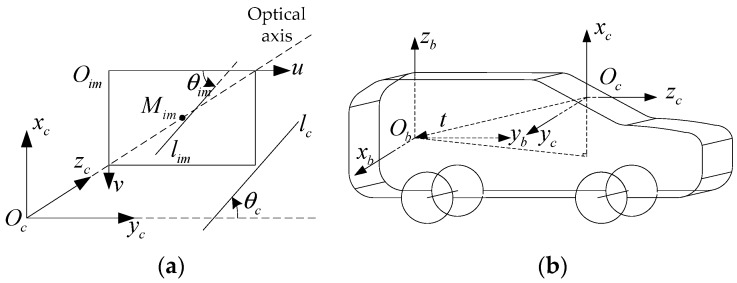
The relationships of the *im*-, *c*-, and *b*-coordinate. (**a**) The relationship between the *im*- and *c*-coordinate; and (**b**) the relationship between *c*- and *b*-coordinate.

From the process of a camera record, as is known to all, we can get the relationship between the *im*-coordinate and the *c*-coordinate, as shown in Figure 2a. The optical axis of the camera superposes with the $z_{c}$ axis and crosses the image plane in the middle point $M_{im}$.

In Figure 2a, the line *l_im_* is in the *im*-coordinate, and its angle feature parameter is *θ_im_*. The projection of $l_{im}$ in *c*-coordinate is $l_{c}$, and the angle feature parameter is $\theta_{c}$. According to the principle of camera imaging, the image plane parallels with the $x_{c}y_{c}$ plane in the *c*-coordinate. So, $l_{c}//l_{im}$. Thus, $\theta_{c}=\theta_{im}$. In other words, the feature angle in the *c*-coordinate is the same as within the *im*-coordinate.

Figure 2b shows the relationship between the *c*-coordinate and *b*-coordinate. From it, the transfer matrix between the *c*-coordinate and *b*-coordinate is given by

(15) $$T_{b}^{c}=\left(\begin{array}{cc} C_{c}^{b} & t\\ 0 & 1 \end{array}\right),C_{c}^{b}=\left[\begin{array}{ccc} 0 & 1 & 0\\ 0 & 0 & 1\\ 1 & 0 & 0 \end{array}\right]\;$$

The vehicle attitude includes the pitching angle, rolling angle, and heading angle. They mean the angle *b*-coordinate rotates around the $x_{b}$, $y_{b}$, and $z_{b}$ axis, respectively. In this paper, we set that the *c*-coordinate is orthogonal to the *b*-coordinate. So, the angle caused by heading and pitching will be not projected onto the *z_c_* axis. Thus, when the vehicle is rolling, *θ_c_* will be changed. Additionally, the pitching and heading of the vehicle cannot change $\theta_{c}$. Thus, we can use $\theta_{c}$ to describe the vehicle-rolling angle. We define the vehicle rolling angle error as $\Delta \theta_{c}$, it is the difference between $\theta_{c}$ and the mean value of ${\bar{\theta }}_{c}$. Thus, the visual attitude error $\Delta A_{c}$ in the *c*-coordinate is derived as

(16) $$\Delta A_{c}=\left[\begin{array}{c} 0\\ \Delta \theta_{c}\\ 0 \end{array}\right]\;$$

Thus, $\Delta A_{c}$ can be translated into the *b*-coordinate as $\Delta A_{b}$, which is expressed as

(17) $$\Delta A_{b}=C_{c}^{b}\Delta A_{c}\;$$

Thus, the visual attitude error in the *n*-coordinate is derived as

(18) $$\Delta A_{n}=C_{b}^{n}C_{c}^{b}\Delta A_{c}\;$$

## 4. The Proposed Fusion Algorithm

**Figure 3:**
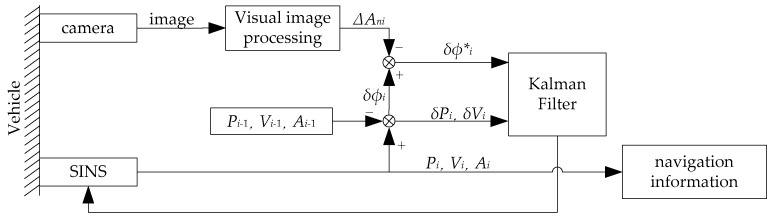
The schematic diagram of inertial-visual integrated navigation system. The strapdown inertial navigation system (SINS) and the camera are fixed on the vehicle. When *i* = 1, the *P*_0_, *V*_0_, and *A*_0_ are the original positions, velocity, and attitude of the vehicle, respectively, in this paper, they are offered by Global Position System (GPS).

In this section, we describe how to fuse the visual attitude error from images with SINS. The inertial-visual integrated navigation system schematic is shown in Figure 3. We fuse the visual attitude error with the SINS attitude error. Then, using Kalman Filter to estimate errors of position, velocity and attitude to improve the accuracy of SINS. In this section, we discuss the algorithm of how to fuse the visual attitude error with SINS.

To fuse the visual attitude error with SINS, the attitude error of inertial-visual integrated navigation system can be obtained as

(19) $$\delta \phi^{*}=\delta \phi -\Delta A_{n}\;$$

Thus, the new measurement vector is expressed as

(20) $$Z=\left[\begin{array}{c} \delta V\\ \delta \phi^{*} \end{array}\right]\;$$

An image is constituted by pixels. The image offered by the camera, which is fixed on the static vehicle, only has slight variations. The line feature in those images is a random value in finite countable values. It is not vailed that applying the white noise model to the inertial-visual integrated navigation system measurement noise. Thus, we cannot fuse the visual attitude error with the SINS directly. It is necessary to find a new model for the inertial-visual integrated navigation system measurement noise.

Ideally, the line feature of images from the camera fixed on static vehicles is the same. That means that the visual attitude error of static vehicles is zero. However, there are some noise sources, like the shuddering of the engine and the actions of the driver and passengers. Thus, $\Delta A_{n}$ can be thought of as the visual attitude measurement noise.

In a known size image, the status number of line feature θ is finite and enumerable. For an appointed line, in all the t frames of the video, line feature θ is only related to the last one. In other words, we define E as the state space of θ. For any $t_{1}<t_{2}<\cdots <t_{p}<t$, there are $\Delta A_{n}^{1}$,$\Delta A_{n}^{2}$,…,$\Delta A_{n}^{p}$∈E. When it is known that $\Delta A_{n}\left(t_{1}\right)=\Delta A_{n}^{1}$, $\Delta A_{n}\left(t_{2}\right)=\Delta A_{n}^{2}$,…,$\Delta A_{n}\left(t_{p}\right)=\Delta A_{n}^{p}$, the condition probability curve is related to $\Delta A_{n}\left(t_{p}\right)=\Delta A_{n}^{p}$, and is not related to $\Delta A_{n}\left(t_{1}\right)=\Delta A_{n}^{1}$,$\Delta A_{n}\left(t_{2}\right)=\Delta A_{n}^{2}$,…,$\Delta A_{n}\left(t_{p-1}\right)=\Delta A_{n}^{p-1}$. That means

(21) $$P\left(\Delta A_{n}\left(t\right)\leq x|\Delta A_{n}\left(t_{p}\right)\leq \Delta A_{n}^{p},\Delta A_{n}\left(t_{p-1}\right)\leq \Delta A_{n}^{p-1},\cdots ,\Delta A_{n}\left(t_{1}\right)\leq \Delta A_{n}^{1}\right)=P\left(\Delta A_{n}\left(t\right)\leq x|\Delta A_{n}\left(t_{p}\right)\leq \Delta A_{n}^{p}\right)\;$$

Thus, the visual attitude measurement noise can be modeled by the first-order Markov Process.

The inertial-visual integrated navigation system measurement noise, $V_{k}$, includes SINS measurement noise and the visual attitude measurement noise. SINS measurement noise is a Gaussian White noise. Additionally, the inertial-visual integrated navigation system is a linear system. Thus, $V_{k}$ also can be modeled by the first-order Markov Process. Thus, $V_{k}$ satisfies the equation as

(22) $$V_{k+1}=\Psi_{k+1,k}V_{k}+\xi_{k}\;$$

where $\Psi_{k+1,k}=e^{-\alpha T}$, α is the inverse correlation time constant of the first-order Markov Process, T is the sample interval, $\xi_{k}$ is white noise with a zero mean and is uncorrelated with $W_{k}$ [20].

From Equation (12), $V_{k}$ can be expressed as

(23) $$V_{k}=Z_{k}-H_{k}X_{k}\;$$

We have combined the Equations (1), (22), and (23) into the right side of Equation (2) here

(24) $$Z_{k+1}=H_{k+1}\left(\Phi_{k+1,k}X_{k}+\Gamma_{k}W_{k}\right)+\Psi_{k+1,k}\left(Z_{k}-H_{k}X_{k}\right)+\xi_{k}\;$$

By rearranging Equation (24), it becomes

(25) $$Z_{k+1}-\Psi_{k+1,k}Z_{k}=\left(H_{k+1}\Phi_{k+1,k}-\Psi_{k+1,k}H_{k}\right)X_{k}+H_{k+1}\Gamma_{k}W_{k}+\xi_{k}\;$$

The measurement matrix and measurement noise vector can then be expressed as

(26) $$Z_{k}^{*}=Z_{k+1}-\Psi_{k+1,k}Z_{k},H_{k}^{*}=H_{k+1}\Phi_{k+1,k}-\Psi_{k+1,k}H_{k},V_{k}^{*}=H_{k+1}\Gamma_{k}W_{k}+\xi_{k}\;$$

Additionally, the new measurement equation becomes

(27) $$Z_{k}^{*}=H_{k}^{*}X_{k}+V_{k}^{*}\;$$

The mean and variance of $V_{k}^{*}$ can be expressed as

(28) $$\left\{ \begin{array}{l} E\left[V_{k}^{*}\right]=0\\ E\left[V_{k}^{*}V_{j}^{*}{}^{T}\right]=\left(H_{k+1}\Gamma_{k}Q_{k}\Gamma_{k}^{T}H_{k+1}^{T}+R_{k}\right)\delta_{kj} \end{array}\right.\;$$

where $V_{k}^{*}$ is white noise with a zero-mean. Thus, the covariance matrix of $V_{k}^{*}$ can be expressed as

(29) $$R_{k}^{*}=H_{k+1}\Gamma_{k}Q_{k}\Gamma_{k}^{T}H_{k+1}^{T}+R_{k}\;$$

$V_{k}^{*}$ is correlated with $W_{k}$, and the correlation coefficient is $S_{k}=Q_{k}\Gamma_{k}^{T}H_{k+1}^{T}$. Therefore, Equations (3), (5), and (7) become

(30) $${\hat{X}}_{k+1}^{*}=\Phi_{k+1,k}{\hat{X}}_{k}+K_{k+1}\left(Z_{k+1}-\Psi_{k+1,k}Z_{k}-H_{k}^{*}{\hat{X}}_{k}\right)\;$$

(31) $$K_{k+1}^{*}=\left(\Phi_{k+1,k}P_{k}H_{k}^{*}{}^{T}+\Gamma_{k}S_{k}\right){\left(H_{k}^{*}P_{k}H_{k}^{*}{}^{T}+R_{k}^{*}\right)}^{-1}\;$$

(32) $$P_{k+1}^{*}=\Phi_{k+1,k}P_{k}\Phi_{k+1,k}^{T}+\Gamma_{k}Q_{k}\Gamma_{k}^{T}-K_{k+1}\left(H_{k}^{*}P_{k}\Phi_{k+1,k}^{T}+S_{k}^{T}\Gamma_{k}^{T}\right)\;$$

## 5. Experiment and Results

**Figure 4:**
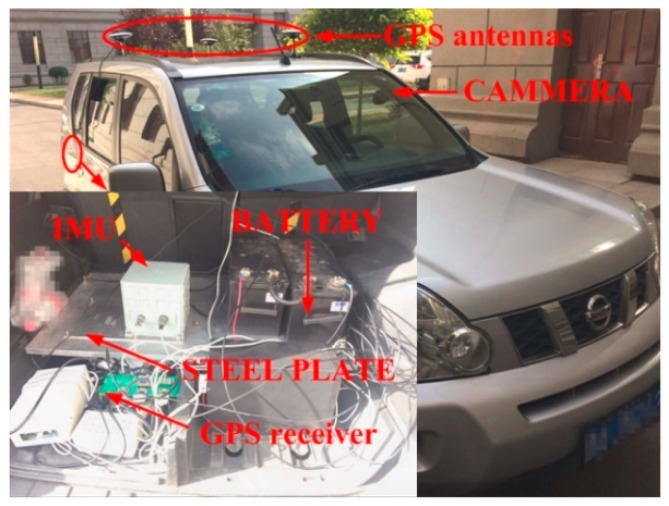
The equipment used in the experiment.

As shown in Figure 4, an experimental system is assembled to evaluate the proposed approach. The system includes an IMU, a camera, a GPS, and a power system on a vehicle. IMU is constituted by a three-axis fiber-optic gyroscope with three accelerometers on each gyro-axis. The camera is a vehicle data recorder. The IMU, GPS receiver, and power system are in the vehicle trunk. The IMU is fixed on the vehicle via a steel plate which is parallel with the under panel of the vehicle. The GPS antennas are on the top of the vehicle. The camera is attached to the windshield. When installing the camera, the Gradienter, and the Vertical marker are used to make sure the optical axis is parallel with the North-East plane of the *n*-coordinate.

During the experiment, the vehicle is started and stopped. In this experiment, the GPS is used to provide a coarse alignment of the position system and to provide a position reference. In the experiment, the frequency of the IMU is 100 Hz, the frequency of the video is 25 Hz, the image resolution is 1920 × 1080, and the static time length is two min.

### 5.1. Camera Information Pre-Processing

**Figure 5:**
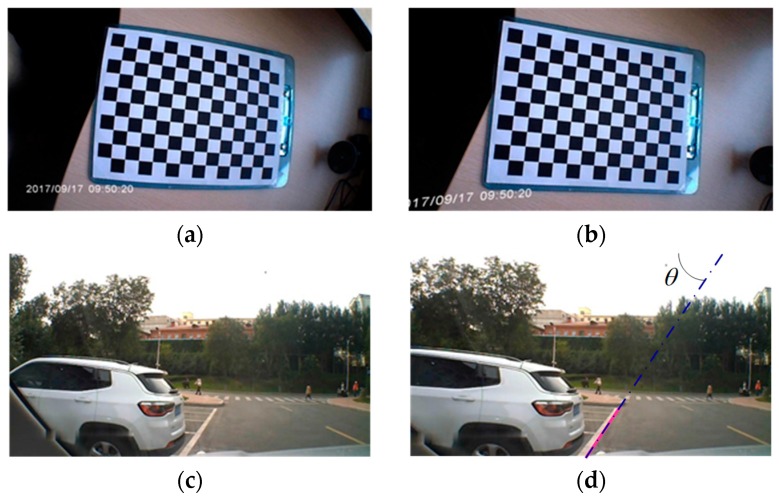
Image processing. (**a**) The original image of the camera; (**b**) the calibrated image of (**a**); (**c**) one of the original images during the experiment; (**d**) the calibrated image of (**c**) and the line feature parameter θ.

The camera used in the experiment is a wide-angle automobile data recorder. As the picture taken from the wide-angle is distorted, the picture needs calibration. Thus, the picture needs pre-process to remove its distortion. We calibrated the camera in a lab environment to get the parameters of the camera using the Lee-method [23]. Camera calibration results are shown in Figure 5a,b. We then used the parameters from the calibration to calibrate the on-road experiment images, as shown in Figure 5c,d. Apparently, the distortion was reduced.

During the experiment, the vehicle stops, the view of the camera is fixed. There are two-lane lines, the white solid line, and the yellow dotted line. We chose the white one as the reference object and extracted its line feature parameters, as shown in Equation (18). As shown in Figure 5d, the pink line is the extraction line, and the blue solid-dotted line is the schematic of the extraction line. After extracting all images in the video during this static experiment, we get θ in Equation (14) of all the images.

Based on the line feature parameter $\theta_{im}$, and the transfer matrixes $C_{c}^{b}$ and $C_{b}^{n}$, calculated the attitude error $\Delta A_{n}$ offered by image processing as shown in Section 3.

### 5.2. Experimental Results and Discussions

In this section, we discuss the experimental results from two aspects, static attitude error and static position estimation. The experimental results prove that the proposed inertial-visual integrated navigation system can improve the accuracy and stability of the navigation system.

**Figure 6:**
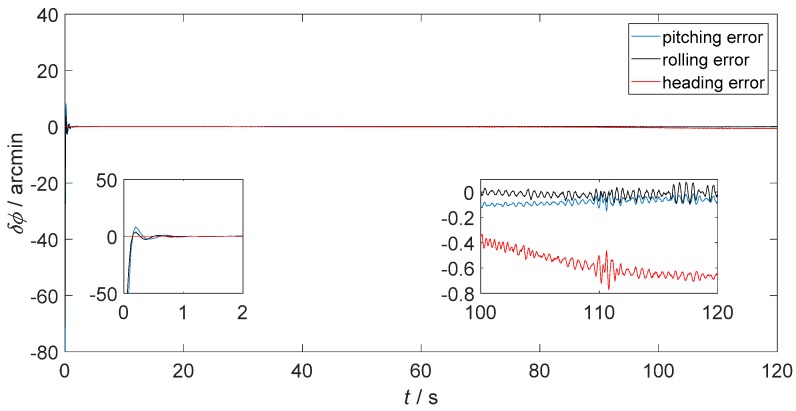
Static attitude error of only-SINS.

**Figure 7:**
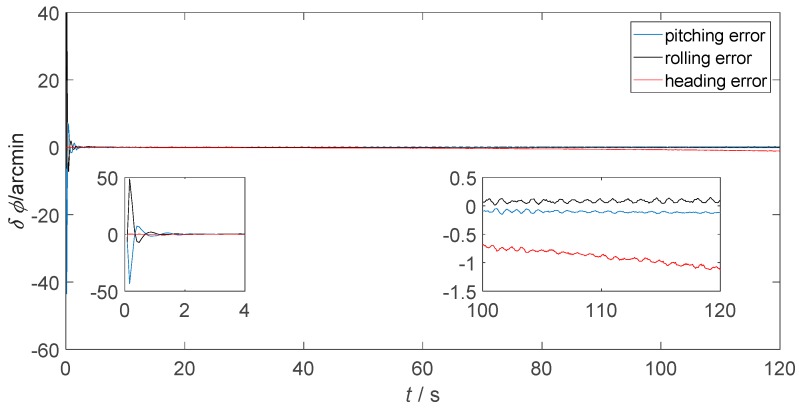
Static attitude error of the integrated navigation system that inertial and visual sensors fused directly.

**Figure 8:**
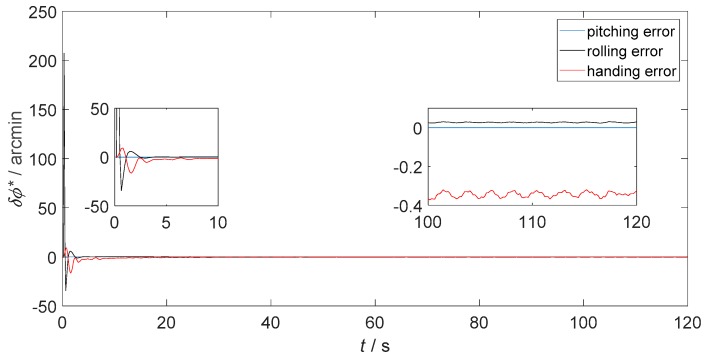
Static attitude error of the integrated navigation system that inertial and visual sensors fused as proposed.

Figure 6 presents the static attitude error of only-SINS. Figure 7 and Figure 8 show the static attitude error of the inertial-visual integrated navigation system that fused directly and as proposed, respectively. The attitude error includes the pitching error, rolling error, and heading error, as shown in the legend. While, the initial alignment times of integrated navigation systems are longer than only-SINS. The initial alignment time of the proposed inertial-visual integrated navigation system meets the requirements of automated vehicle applications. Besides, the integrated navigation system has a longer initial alignment time that resulted from the data rate of the camera being lower than that of the IMU. Thus, using high data rate cameras is a useful method to reduce the initial alignment time of the integrated navigation system [24].

The attitude errors from 100 s to 120 s are shown in the enlarged inset pictures of Figure 6, Figure 7 and Figure 8. For only-SINS, the heading error was increased, up to −0.65 arcmin at 120 s. For the direct inertial-visual integrated navigation system, the heading error was increased too, up to −1.20 arcmin at 120 s. It confirms that the white noise model does not fit to the inertial-visual integrated navigation system measurement noise, while the attitude errors of the proposed model are stable, as shown in Figure 8. The heading error keeps at −0.35 arcmin in the enlarged picture. The heading error of the proposed integrated method is more stable than the only-SINS and the direct integrated method during, from 20 s to 120 s. It is decreased by 46.15% compared to the heading error of only-SINS at 120 s. The Figure 6, Figure 7 and Figure 8 show that the proposed inertial-visual integrated navigation system improves the accuracy and stability of the navigation system.

**Figure 9:**
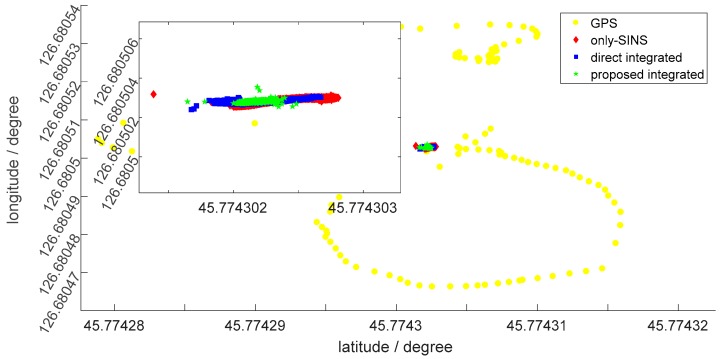
The position estimation of GPS, only-SINS, direct inertial-visual integrated navigation system, and proposed inertial-visual integrated navigation system.

Figure 9 shows the static position, during 120 s, by the latitude and longitude of the GPS, only-SINS, direct inertial-visual integrated navigation system, and the proposed inertial-visual integrated navigation system, as shown in the legend. The enlarged inset picture indicates the position of the only-SINS and integrated navigation system. As shown in Figure 9, the position estimation range of the proposed inertial-visual integrated navigation system is more concentrated than the only-SINS and direct inertial-visual integrated navigation system. For GPS, the amplitude variation of latitude and longitude are 4 × 10^−50^ and 8 × 10^−50^, respectively. The GPS position can prove that the position estimations of the other three navigation modes is receivable. For the only-SINS, the amplitude variation of latitude and longitude are 1.4236 × 10^−60^ and 6.3310 × 10^−70^, respectively. For the direct inertial-visual integrated navigaiton system, the amplitude variation of latitude and longitude are 9.9625 × 10^−70^ and 6.7123 × 10^−70^, respectively. The position range is not obvious difference with only-SINS. Thus, the direct integrated method cannot improve the accuracy of the navigation system. For the proposed inertial-visual integrated navigaiton system, the amplitude variation of latitude and longitude are 8.3885 × 10^−70^ and 3.5869 × 10^−70^, respectively. The position estimation of the proposed inertial-visual integrated navigation system is more stable than that of only-SINS and direct inertial-visual integrated navigation system. It also can be reflected by the position standard deviation, as listed in Table 1. Figure 9 shows that the proposed inertial-visual integrated navigation system improves the accuracy of the position estimation.

Table 1 lists the position standard deviation of GPS, only-SINS direct inertial-visual integrated navigation system, and the proposed inertial-visual integrated navigation system. It shows the standard deviation of the proposed inertial-visual integrated navigation system is lower than only-SINS and direct inertial-visual integrated navigation systems. Compared with only-SINS, standard deviation of the proposed inertial-visual integrated navigation system decreases by 69.34% and 21.27% of latitude and longitude, respectively. The latitude and longitude standard deviation of the proposed inertial-visual integrated navigation system are 35.59% and 72.99% of the direct inertial-visual integrated navigation system. The data in Table 1 verifies that the inertial-visual fusion method can improve the stability of the navigation system.

## 6. Conclusions

In this paper, it is the first time that the inertial-visual integrated navigation system, which combined the line feature in the image with SINS via KF, has been proposed. The experimental results show that the proposed inertial-visual integrated navigation system improves the accuracy and reliability of the navigation system prominently. In the meantime, the inertial-visual integrated system can keep the autonomy of the SINS. This method provides a high accuracy inertial-visual integrated navigation system and fills the static condition of an automated vehicle. At the same time, it lays a theoretical foundation for the research of the proposed method in dynamic situation.

The future work plan includes the proposed inertial-visual integrated system used on automated vehicle moves, like straight driving and making a turn. For the information fusion algorithm, the improved Unscented Kalman Filter (UKF) will be used to reduce the computational load and improve the robustness of the KF [25,26]. Additionally, the line feature recognition algorithm will be perfected to improve the accuracy, stability, and reliability of the navigation system.

## Figures and Tables

**Table 1:** The Position Standard Deviation of GPS, only-SINS direct inertial-visual integrated navigation system and proposed inertial-visual integrated navigation system.

	Latitude	Longitude
GPS	8.490414716838 × 10^−6^	2.278273360829 × 10^−5^
only-SINS	2.367825000248 × 10^−7^	1.528532246718 × 10^−7^
direct integrated	2.039732103084 × 10^−7^	7.745355384118 × 10^−8^
proposed integrated	7.260776501218 × 10^−8^	5.653344095192 × 10^−8^
